# Development of Bioactive *Opuntia ficus-indica* Edible Films Containing Probiotics as a Coating for Fresh-Cut Fruit

**DOI:** 10.3390/polym14225018

**Published:** 2022-11-18

**Authors:** Tatsaporn Todhanakasem, Pratana Boonchuai, Pavarunya Itsarangkoon Na Ayutthaya, Rachit Suwapanich, Bongkot Hararak, Bo Wu, Briana M. Young

**Affiliations:** 1 School of Food Industry, King Mongkut’s Institute of Technology Ladkrabang, Chalong Krung 1 Alley, Lat Krabang, Bangkok 10520, Thailand; 2 National Metal and Materials Technology Center (MTEC), National Science and Technology Development Agency (NSTDA), Khlong Luang 12120, Thailand; 3 Biomass Energy Technology Research Center, Biogas Institute of Ministry of Agriculture and Rural Affairs, Renmin Rd. S 4-13, Chengdu 610041, China; 4 Department of Medical Microbiology and Immunology, School of Medicine, University of California Davis, One Shields Ave, Davis, CA 95616, USA

**Keywords:** *Opuntia ficus-indica*, *E. faecium*, edible active film, mechanical strength

## Abstract

Bioactive edible films have received more attention in recent years as a method for food preservation with value-added functions. The aim of this study was to develop a bioactive edible film containing mucilage of cactus (*Opuntia ficus-indica*) and incorporating the probiotic strain *Enterococcus faecium* FM11-2 as an active component to promote consumer health benefits. *Opuntia ficus-indica* is rich in nutritional and bioactive compounds and the abundance of this cactus makes it attractive for food applications. Mucilage of *Opuntia ficus-indica* contained 0.47 ± 0.06 mg/g total sugar, 0.33 ± 0.06 mg AGE/mL phenolic content, 0.14 mg/ mL vitamin C, and possessed 35.51 ± 1.88% DPPH scavenging activity. The edible film that was developed exhibited the following characteristics: thickness of 0.02–0.11 mm, percent moisture content 0.19–0.24%, water solubility 30.66–59.41% and water vapor permeability of 0.15–1.5 g·mm/m^2^·min·kpa, while the range of the variation depended on the type of plasticizer used (either sorbitol or glycerol). The addition of sorbitol in the film provided the maximum mechanical strength based on the evaluation of tensile strength, Young’s modulus and elongation at break (44.71 ± 0.78 MPa, 113.22 ± 0.23 MPa and 39.47 ± 0.61%, respectively). The optimal formulation of the edible film, according to the physicochemical, physical and maintenance of fresh-cut apple slices, contained cactus mucilage, gelatin, glycerol and a probiotic. The incorporation of a probiotic into the cactus film created a bioactive edible film that could provide a health benefit. While improvement is needed to maintain the survival rate of the probiotic, this work presents an exciting method for furthering the study of food preservation with edible films.

## 1. Introduction

Active food packaging films have been developed to be eco-friendly, enhance product shelf life and meet the demand for healthier foods. Recently, there have been dramatic improvements in the development of edible films and biodegradable films due to the growing interest in reducing environmental pollution caused by global plastic usage. It has been suggested that food plastics and wrappers account for over half the world-wide plastic usage. Edible films act as a barrier between the covered product and the surrounding medium to delay the migration of moisture, gases and lipids. More recently, active edible films have been developed to carry more additives such as nutrients, flavors, antimicrobials, oxygen scavengers, antioxidants, antimicrobials, ethylene scavengers, and moisture absorbers [1]. The principle components for producing edible/biodegradable films are film forming biopolymers, such as carbohydrates, proteins, a solubilizing medium (water, ethanol, etc.) and plasticizers [2]. The major advantages of the use of such components is that they provide moisture resistance, a water-soluble nature, gelling properties, good thermal and mechanical properties, antimicrobial activity, heat-based sealing, and are flexible and colorless. [3].

*Opuntia ficus-indica* (OFI) is the most cultivated edible cactus in the world. The mucilage of OFI is a polysaccharide with a molecular weight in the order of 2.3 × 10^4^ to 4.3 × 10^6^ Da and is rich in D-galactose, L-arabinose, D-xylose, and L-rhamnose, as well as D-galacturonic acid [4,5]. It is also rich in polyphenols, vitamins, polyunsaturated fatty acids and amino acids, all of which have been isolated through the use of a large panel of extraction methods. It has been used in traditional folk medicine because of its role in treating a number of diseases and conditions and has been shown to have properties that are anti-inflammatory, antioxidant, and antimicrobial. It has been used to modulate hypoglycemic effects, inhibit stomach ulceration and may have neuroprotective effects. Additionally, cactus mucilage has been used for treating diabetes, burns, bronchial asthma and indigestion in many countries throughout the world, and beneficial outcomes are attributed to its antioxidant effects [6,7]. Some of the antioxidant compounds that have been identified in OFI are flavonoids and phenolic acids. OFI also contains considerable amounts of vitamins, mainly ascorbic acid, vitamin B, and α-tocopherol. OFI mucilage is a complex polysaccharide, can be used as dietary fiber and has the capacity to absorb large amounts of water by dissolving, dispersing and forming viscous or gelatinous colloids [8]. There are uses for cactus mucilage in food, cosmetics, pharmaceutical and other industries. There has already been progress in using the mucilage to create edible coatings, which are an interesting alternative to extend the shelf-life of fresh-cut foods, reduce microbial contamination, provide resistance to gas exchange, delay moisture loss, and prevent unwanted darkening reactions [9]. Mucilage from Mexican *Opuntia ficus-indica* has been developed as an edible film, with the addition of polymers such as pectin and glycerol as a plasticizer to help the polymer matrix formation [10]. Previous work reported that Breba figs and strawberries coated with a mucilaginous solution of OFI exhibited prolonged shelf life and showed reduced weight loss while maintaining brightness, visual appearance and firmness; however, the coating did not prevent microbial decay of the figs [11,12]. Additionally, edible film coatings based on spineless cactus mucilage reduced dehydration and maintained visual and sensory qualities of yam slices [13].

Recently, integration of probiotics into bioactive edible food packaging films has been extensively studied, with a focus toward maintaining food quality for health benefit purposes. Probiotics, as well as many other active compounds, have been incorporated into biopolymeric matrices to develop active/bioactive food packaging materials as an alternative method for controlling pathogenic microorganisms and improving food safety, with the added potential to favor consumer health [14]. When probiotic microorganisms are incorporated into foods, they must be able to survive passage through the gastric juices of the digestive tract and then successfully proliferate in the gut. An interesting option for delivery of probiotics is to use an edible food packaging matrix that protects the bacteria and favors their survival. Previous work has shown that edible polymers can act as a carrier and protect probiotic viability as the bacteria travel through the gastrointestinal tract [15]. Pullulan and various starches (from potato, tapioca, and corn) have been used to develop novel edible films incorporated with a mix of probiotic cultures (*Lactobacillus reuteri* ATCC 55730, *L. rhamnosus* GG ATCC 53103, and *L. acidophilus* DSM 20079) [16]. In addition, Rodriguez et al. (2018) evaluated the shelf life of fresh-cut yacon cubes wrapped in edible coatings based on linseed mucilage, alginate, and fructo-oligosaccharide containing *Lactobacillus casei* LC-01. They found that the use of these films helped preserve the physicochemical parameters of the vegetable by reducing weight loss and darkening [17].

The aim of our current study was to evaluate formulations of edible films composed of cactus mucilage from OFI, plasticizers, a probiotic (*E. faecium* FM11-2 from fermented chicken isolates) and gelatin. The physicochemical and mechanical properties of the film were analyzed to determine the best formulations. We further evaluated the newly developed bioactive edible film as a coating on fresh-cut apple slices and determined the freshness, weight loss and probiotic survival rates. This study provides insight for the further exploration of ingredients and physicochemical/mechanical properties for the development of bioactive edible coatings. These finding can now be applied to develop more variations in films for use with fresh-cut fruits and vegetables for health benefits and food preservation in the future.

## 2. Materials and Methods

### 2.1. Cactus Mucilage Extraction

*Opuntia ficus indica* were purchased from Chang Phueak district, Chiang Mai province, Thailand. The size of the leaves ranged between 30 and 40 cm in length, with an age of approximately 2 years. Fresh pads were washed with distilled water and the thorns were removed. The outer layer was removed, while leaving the interior white flesh intact. The extraction of mucilage from cactus pads of OFI was performed according to the method of Rim et al. (2018) and Otálora et al. (2021) with modification [18,19]. The interior white flesh was cut into small pieces and placed in a 1000 mL beaker with distilled water 1:2 *v/v* (flesh: distilled water). The mixture was left at room temperature for 24 h and then the extract was filtered through nylon cloth. The extract was centrifuged at 12,880× *g* force at 4 °C for 30 min to remove the remaining particles. Subsequently, 95% *v/v* ethanol was added to the extract at a ratio of 1:2 *v/v* (extract: 95% *v/v* ethanol) for one night at 4 °C to precipitate the mucilage. The ethanol was then completely evaporated in a water bath set to 50 °C. The residue was collected in a 1000 mL Duran flask for further application.

### 2.2. Analysis of the Composition of Cactus Mucilage

#### 2.2.1. Total Sugar Content

Total sugar content of the cactus mucilage was evaluated using the phenol-sulfuric method, which is a simple and rapid colorimetric method that uses Absorbance at OD_490_ using a spectrophotometer [20]. The concentration range of the glucose standard solution was 0–0.2 mg/mL. A 10-fold dilution of the cactus mucilage was evaluated and compared to the glucose standard curve to obtain the total sugar content.

#### 2.2.2. Total Phenolic Content

The total phenolic content of cactus mucilage was determined using the Folin Ciocalteu method from Rim et al., 2018, with modification [18]. The standard curve was prepared using a gallic acid (Sigma-Aldrich, St. Louis, MO, USA) solution at concentrations of 0–0.3 mg/mL. The reactions were performed by pipetting 500 µL of each concentration of gallic acid solution into test tubes, followed by the additions of 5 mL of Folin Ciocalteu reagent (Sigma-Aldrich, St. Louis, MO, USA) (10%) and 4 mL of 7% *w/v* sodium carbonate solution. The reactions were mixed after the addition of each reagent and then incubated for 15 min in the dark. The absorbance was monitored at OD_765_. To measure test samples, aliquots of 500 μL of mucilage were mixed with 5 mL Folin Ciocalteu reagent and 4 mL of 7% *w/v* sodium carbonate following the standard protocol. The absorbance was measured at 765 nm and the results were expressed as mg of gallic acid equivalent per g of mucilage (mg GAE/g MUC).

#### 2.2.3. Vitamin C Content

Vitamin C content was determined according to the method of Nantawan et al. (2017) with some slight modifications [21]. Cactus mucilage (2 mL) was added to an Erlenmeyer flask containing 100 mL of distilled water. Next, 5 mL of 1% *w/v* starch solution was added. The mixture was shaken and titrated with an iodine solution to the end point. The volume of iodine solution used was recorded and was used to calculate the vitamin C content using the triangular law calibration. The concentration of 1 mg/mL of ascorbic acid (Sigma Aldrich, St. Louis, MO, USA) solution is equivalent to the use of 13.6 mL of iodine solution for the titration. If 1 mL of iodine solution was used, the content of ascorbic acid was 0.074 mg/mL.

Therefore, the ascorbic acid content of the cactus mucilage was calculated from:

*A* = *V*_*I*2_ × 0.08

where:

*A* is the amount of ascorbic acid in the sample solution (mg)
*V*_*I*2_ is Volume of iodine used for titration (mL)

#### 2.2.4. Radical Scavenging Effect

Free radical scavenging was determined using the DPPH radical scavenging method. First, 0.1 mM of methanol-2,2-diphenyl-1-picrylhydrazyl (DPPH) (Sigma-Aldrich, St. Louis, MO, USA) was prepared in methanol and the absorbance was measured with a spectrophotometer at 517 nm for the determination of the percentage inhibition. Then, 0.5 mg/mL cactus mucilage was prepared in distilled water and 1 mL was pipetted into test tubes, followed by the addition of 2 mL of 0.1 mM of DPPH solution. The reaction was mixed well and set aside in the dark for approximately 30 min, after which the absorbance was measured at OD_517_. Experiments were repeated three times, and the precent inhibition was calculated using the following equation:

% *Inhibition* = (*A_DPPH_* − *A_Sample_*/*A_DPPH_*) × 100

where

*A_sample_* is the absorbance of the *sample* + *DPPH*
*A_DPPH_* is the absorbance of *DPPH*

### 2.3. Preparation of Probiotic Cells

*E. faecium* FM11-2 was isolated from Thai fermented chicken in our previous work [22]. The strain was streaked on MRS (deMan, Rogosa, Sharpe, Oxoid, Basingstoke, UK) agar and incubated at 37 °C for 48 h prior to culture in MRS broth for another 48 h until the OD_600_ reached approximately 1.0. The suspended culture was then ascetically transferred into eppendorf tubes (1 mL per tube) and centrifuged at 2060× *g* force at 4 °C for 5 min to collect the cell pellets. The supernatant was discarded, and the cell pellets were washed twice (1 mL per wash) with 0.85% sodium chloride (NaCl) solution and resuspended at a concentration of 1.2 × 10^8^ CFU/mL prior to incorporation into the film.

### 2.4. Preparation of Probiotic Cactus Film

Films were prepared as described by Rim et al. (2018) with some modification [18]. Cactus mucilage, gelatin from porcine skin (Sigma Aldrich, St. Louis, MO, USA), distilled water, and plasticizer (glycerol (Sigma Aldrich, St. Louis, MO, USA) or sorbitol (Sigma Aldrich, St. Louis, MO, USA) were mixed with continuous agitation using a magnetic stirrer at 70 °C for approximately 10 min according to the formula in Table 1 to ensure homogenous mixing. The prepared mixtures were cast onto plastic Petri dishes (total volume of 15 mL) and dried in a ventilated oven at 45 °C for 24 h to form a film. The resulting films were stored in a desiccator at 25 °C and 53% RH for 48 h prior to testing. RH was monitored using a psychrometer based on a dry and a wet temperature sensor. To incorporate the probiotic (*E. faecium* FM11-2), the mixtures were aseptically prepared as described above, after which the solution was cooled to 37 °C and the probiotic was added at a concentration of 1.2 × 10^8^ CFU/mL. The films were dried at 35 °C for 24 h under aseptic conditions. Additionally, 50 mL of the solution was kept for dipping fresh cut apple for further analysis.

### 2.5. Characterization of Film Physical Properties

#### 2.5.1. Film Thickness

The thickness of the film was evaluated using a micrometer (Mitutoyo, Kawasaki, Kanagawa, Japan) with measurements taken at 5 arbitrary locations and the mean value of the films was calculated [23].

#### 2.5.2. Moisture Content

The moisture content of the films was determined using a modification of the protocol by Rim et al. (2018) [18]. The film samples were cut into pieces with an initial weight (*m_i_*) of 0.5 g and then oven dried at 105 °C until the weight was stable to obtain the final weight (*m_f_*). The moisture content of the film was calculated based on the formula:

*%MC* = *m_i_* − *m_f_/m_i_* × 100

where %*MC* is the percent moisture content of the film, *m_i_* is the initial weight of the film (g) and *m_f_* is the weight of the film after curing to a constant weight (g).

#### 2.5.3. Film Solubility

Film solubility was analyzed according to the method of Jouki et al. ( 2013) with some modifications [24]. Film samples of 0.5 g were oven dried at 105 °C until constant weight was achieved (*m_i_*) and then cooled to room temperature. The samples were then submerged in 50 mL of distilled water at room temperature for 30 min. The undissolved matter was collected from the liquid and oven dried at 105 °C until the weight was constant (*m_f_*). The water solubility of the film is reported as a percentage of water solubility (% water solubility) and calculated using the equation:

*%WS = m_i_* − *m_f_/m_i_* × 100

#### 2.5.4. Water Vapor Permeability

Water vapor permeability of the film was analyzed according to the ASTM E96/E96M (2015) standard method with some modifications. The experiment was conducted using the desiccant method. The film samples were sealed on an open cup containing 15 g of pre-dried silica gel and incubated at room temperature (35 °C and 60% RH) until the weight was stable [25]. Water vapor transmission is measured by weight gain (Weight change as function of time) and calculated using a linear regression equation (R^2^ > 0.99) and water vapor permeability (g·mm/m^2^·min·kPa):

*WVP = WVTR* × *X*/Δ*p*

where:

*WVTR* (g/m^2^) is the water vapor permeability rate. It is obtained as the slope (g/min) divided by the transmission area (m^2^).
*X* (mm) is the film thickness.
Δ*p* (kpa) is the difference in water vapor pressure between the films (Δ*p* = *p*(RH_2_ − RH_1_) = 337.60 where *p* is the saturation pressure of water at 35 °C, RH_2_ = 60% and RH_1_ = 0%)

#### 2.5.5. Mechanical Properties

Mechanical testing of the film was performed by Texture Analyzer model TA-XT plus C (Stable Micro Systems, Godalming, UK) to assess tensile strength, the elongation at break, and the Young’s modulus (stress-strain) in accordance with the ASTM D882 (2001) standard test method. Each film sample was cut to a size of 15 mm × 60 mm and a test speed of 100 mm/min was used. The values of tensile strength, elongation at break and Young’s modulus are displayed using the program Exponent.

### 2.6. Properties of Film Coating on Fresh-Cut Apple

The apple was cut into pieces and soaked in 1.5% *w/v* saline solution for 10 min to prevent the formation of melanin that can cause a browning reaction on apple pulp. The samples were then dipped in film formulations 5 and 6 containing probiotics (Table 1). The dipped apples were left to dry on a sieve for 10 min. The samples were then refrigerated at 4 °C and the weight loss, analysis of the change in appearance and the viability of the probiotic were analyzed on days 0, 2, 5 and 7. To determine the survival of probiotics on the coated apples, 10 g of the coated apple sample was placed in a bag containing 90 mL of 0.85% saline. The sample was dissociated with a Stomacher, and then diluted to the desired level. The spread plate technique was used to inoculate MRS Agar medium and plates were incubated at 37 °C for 2 days. The colonies formed on the medium were reported as log CFU/g.

### 2.7. Statistical Analysis

All experiments were performed in triplicate and the data was statistically analyzed by ANOVA with Duncan’s multiple range to compare between the different treatments.

## 3. Results and Discussion

The complex nature of cactus mucilage makes it a valuable resource for nutritional and health benefits [26]. Previous studies have found that ethanol facilitates the extraction of phenolic compounds and antioxidant compounds from OFI, so we used this methodology to direct our studies. Our results determined levels of total sugar, phenolic content, vitamin C and DPPH radical scavenging for the extracted cactus mucilage, as shown in Table 2. Preservation of active groups, such as phenolic acids, is key in creating a useful biological packaging film and their antioxidant potential is involved in many health benefits such as prevention of inflammation, cardiovascular dysregulation, and neurodegenerative diseases [27]. Additionally, antioxidant activity can depend on the presence of vitamin C, therefore we evaluated the DPPH radical scavenging activity of the extracted cactus mucilage. Our results indicate that our extraction method preserved nutritional value and active compounds that may promote health in combination with the bioactive edible film. 

**Figure 1:**
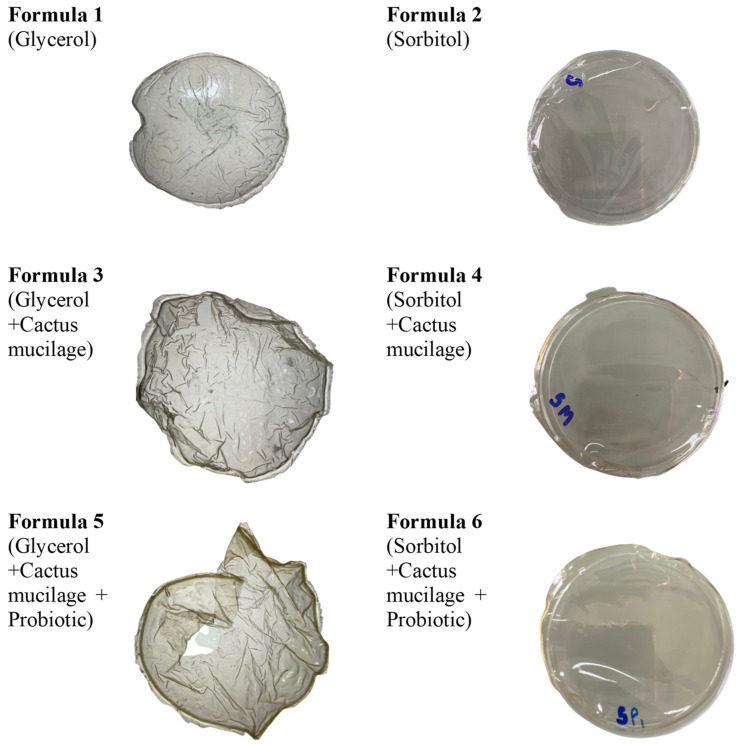
Film characteristics among the 6 formulas.

The appearance of the edible films among the 6 formulas (Figure 1) differed based on the plasticizer used. Both glycerol and sorbitol are suggested as good plasticizers for gelatin, as they can reduce intermolecular hydrogen bonding in gelatin and increase intermolecular spacing. It is postulated that the high molecular weight of sorbitol creates larger intermolecular spacing and leads to better film flexibility [28]. In our study, the films with glycerol as a plasticizer were rough and brittle, whereas the films with sorbitol were smooth and tough. The brittleness of film plasticized with glycerol can be explained due to its low molecular weight of 92 g/mol, in comparison with 182 g/mol for sorbitol [29,30].

Physicochemical properties determine the ability of the edible films to maintain the integrity of packed products. Critical parameters are the strength of the material, including the film thickness, percent moisture content, percent film solubility and WVP [31]. Film formula 6 contained cactus mucilage, gelatin, sorbitol and probiotic and was the thickest of our formulations (thickness 0.11 ± 0.04 mm) (Table 3). The addition of the probiotic and sorbitol had a significant effect on the thickness of the edible film, and it has been shown that film thickness is mostly related to the concentration of solid matter and the viscosity of solutions. Additionally, there are reports that the addition of a probiotic and the number of solutes incorporated could increase the solid content of the film forming solution [32,33]. Furthermore, with a constant amount of gelatin and plasticizer, the film thickness of high-viscosity solutions was thicker than that of low-viscosity solutions because of the influence of water vapor permeability [34,35]. In the previous work, films formulated with OFI mucilage and additives including glycerol, polyethylene and glycol-oleic acid, exhibited increased film thickness (to 0.222 mm) [13]. The moisture content and film solubility are important factors in determining the melting of edible films in the mouth [16]. In the present study, the presence of probiotics and the addition of either of the two different plasticizers caused no significant effect on film moisture. Similar results were found in previous studies with the application of probiotics [36]. Moisture content of the films composed of glycerol were in the range of 0.21–0.36%. In the presence of a constant amount of polymer, the films composed of glycerol generally retained more moisture than the films composed of sorbitol (range of 0.19–0.21%). This finding correlated well with the results in the literature [37]. While the combination of probiotic and sorbitol seemed to be the optimal formula for the edible film based on film thickness and percent moisture content, the percent film solubility was only 30.66%. Therefore, the probiotic with cactus mucilage using glycerol as a plasticizer would be a more suitable edible film due to higher solubility and less cohesion in the polymer matrix. This should lead to a preferable mouth feel. Although edible films and coatings may not completely replace external food packaging, they can provide a good barrier to oxygen and water, which can prolong food stability by reducing the rates of moisture and gas transfer between food and the surrounding environment [31]. Previous studies have shown that moisture plays a key role in food spoilage, and, therefore, to be a useful component for food storage, edible films must create a low enough water vapor barrier to circumvent the issue [38,39]. The film must not only exclude atmospheric water from the product, but also prevent water loss from the food that is wrapped [40,41]. In our study, bioactive edible films made with sorbitol exhibited a higher WVP value (0.16–1.50 g·mm/m^2^·min·kpa) when compared with those made with glycerol (0.05–0.31 g·mm/m^2^·min·kpa). This higher permeability and moisture loss may make sorbitol a less desirable plasticizer for food preservation (Table 3). Additionally, of the two different plasticizers tested (glycerol and sorbitol), sorbitol films were less fragile and probably not applicable for bioactive edible films (due to mouth feel) (Figure 1). Percent film solubility when glycerol was used as a plasticizer was approximately 53–61%, while that of sorbitol was 31–45%. The higher solubility is important because the coating would be consumed with the product as an edible matrix [41]. Our results indicate glycerol-based plasticizers are more applicable for use in formulations of OFI edible films.

**Figure 2:**
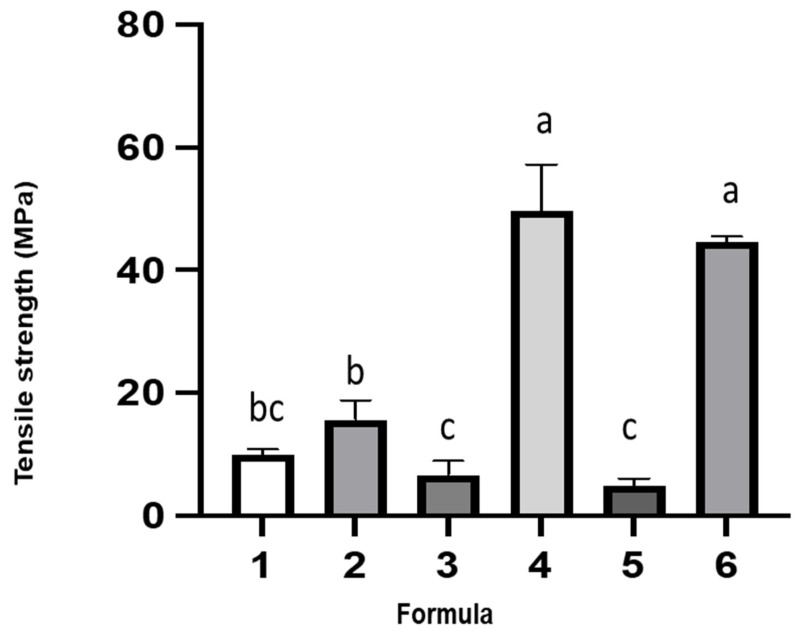
Tensile strength (MPa) of the 6 film formulas. The different letters represent a significant difference of *p* < 0.05. The error bar indicates the standard deviation (*n* = 3).

**Figure 3:**
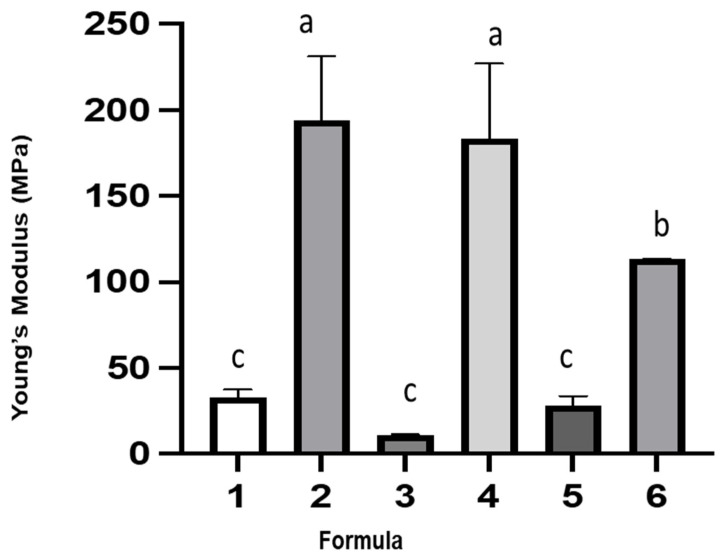
Young’s Modulus (MPa) of the 6 film formulas. The different letters represent a significant difference of *p* < 0.05. The error bar indicates the standard deviation (*n* = 3).

**Figure 4:**
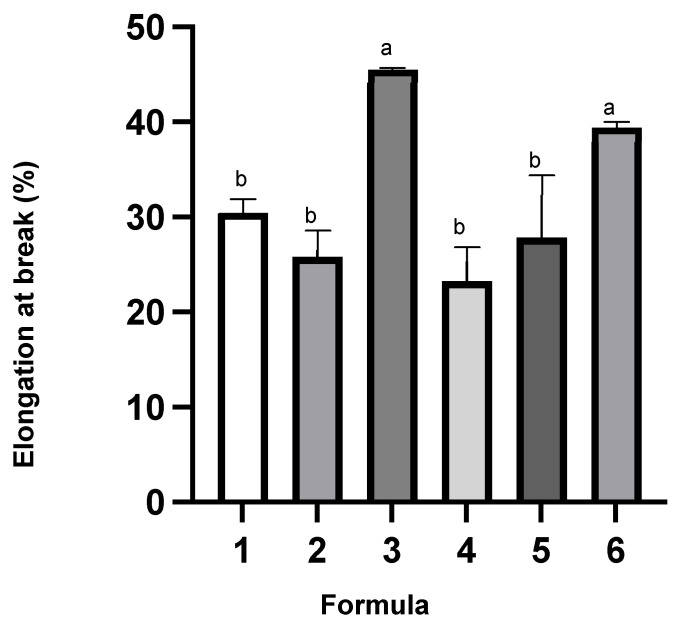
Percent elongation at break (%) of the film. The different letters represent a significant difference of *p* < 0.05. The error bar indicates the standard deviation (*n* = 3).

Tensile properties of the films, including tensile strength, Young’s modulus and elongation at break, were measured and presented in Figure 2, Figure 3 and Figure 4, respectively. The use of either glycerol or sorbitol as a plasticizer for neat gelatin had no significant difference on tensile strength. However, incorporation of sorbitol enhanced the tensile strength when gelatin was mixed with cactus mucilage. The film (formula 4) prepared from mixtures of gelatin, cactus mucilage, and sorbitol had the highest tensile strength (50 MPa), while the addition of a probiotic had no significant effect (45 MPa). The same trend was noticed in the films plasticized with glycerol. From this we conclude that the addition of probiotic had no significant effect on the tensile strength. Young’s modulus of the films containing glycerol was below 40 MPa, while that of the films containing sorbitol increased significantly to a value higher than 100 MPa. Young’s modulus of the film prepared from the mixture of gelatin, cactus mucilage and sorbitol (formula 4) was 183 MPa, and it dropped significantly to 113 MPa when probiotic was added (formula 6). The increase in tensile strength and Young’s modulus might be due to the presence of more hydroxyl groups in sorbitol compared to glycerol. Interaction between sorbitol and other components such as gelatin and cactus mucilage extracts could occur through hydrogen bonding. The highest elongation at break of 45% was found for the film prepared from the mixture of gelatin, cactus mucilage and glycerol (formula 3) and the elongation decreased to 23% when probiotic was incorporated (formula 5). The film prepared from gelatin, cactus mucilage and sorbitol (formula 4) exhibited 23% elongation at break, which increased to 39% when probiotic was added (formula 6). Overall, these results suggest that the addition of sorbitol tended to provide better mechanical properties, in comparison to glycerol. Film formula 6 exhibited the most robust mechanical properties based on tensile strength, Young’s modulus and elongation at break (44.71 ± 0.78 MPa, 113.22 ± 0.23 MPa and 39.47 ± 0.61%, respectively). These characteristics indicate that this film might be better suited for food wrapping applications, rather than as an edible film component.

**Figure 5:**
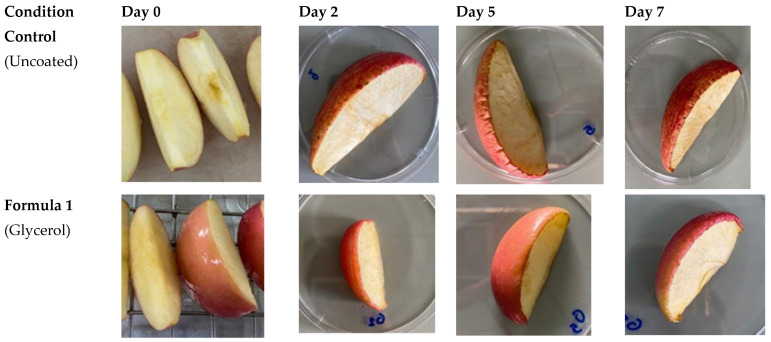

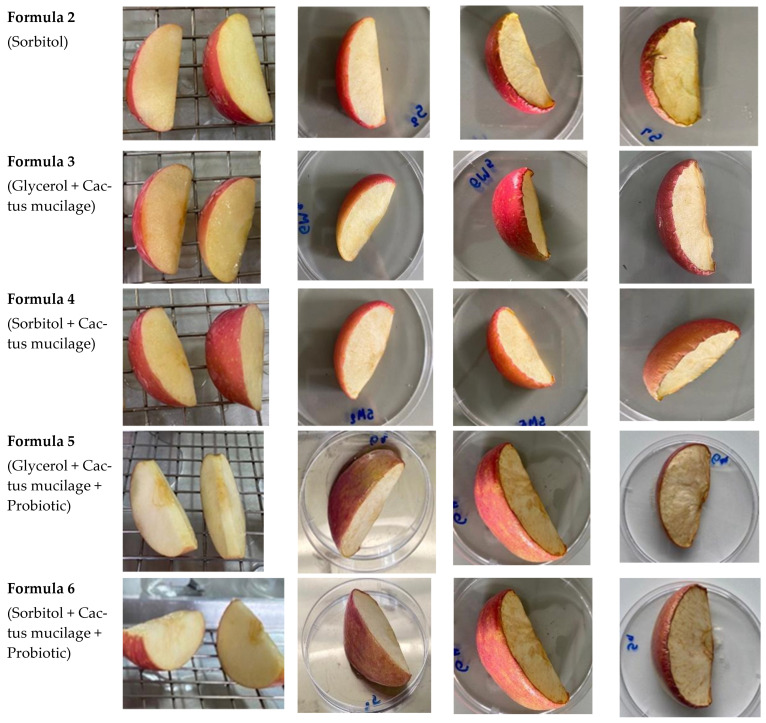
The appearance of fresh cut apple slices dipped in edible coating compared to the control (uncoated) after refrigeration at 4 °C on days 0, 2, 5 and 7.

We also evaluated the influence of the edible coatings on fresh-cut apple slices based on physicochemical parameters including the freshness and weight loss after refrigeration at 4 °C on days 0, 2, 5 and 7. In vegetables, weight reduction during storage occurs by the loss of dry matter through respiration and by the loss of water due to transpiration. Thus, the physiological activity of fruits/vegetables during storage promotes the degradation of compounds, reduction in appearance, and quality loss which can cause rejection of the product by consumers [42]. Our results (Figure 5) indicate that control samples without an edible coating exhibited characteristics which make them unlikely for consumption. In contrast, the samples coated with any of the tested formulas still partially maintained their freshness and extended the shelf life of the fresh-cut apples.

**Figure 6:**
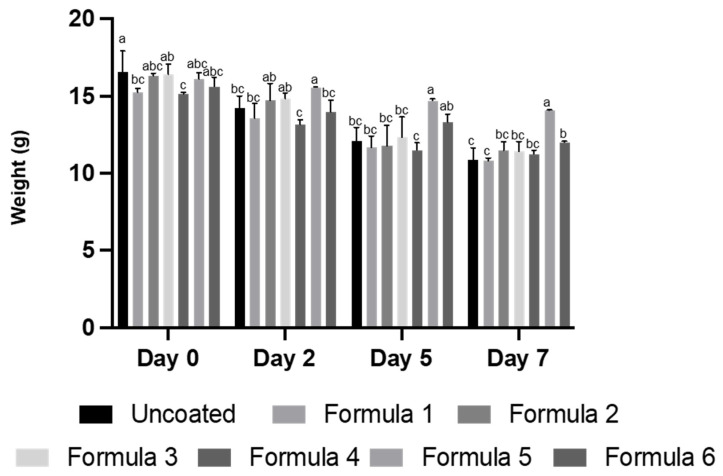
Weight (g) of coated fresh cut apple slices compared to control (uncoated) after refrigeration at 4 °C on days 0, 2, 5 and 7. The different letters represent a significant difference of *p* < 0.05.

In this study, the weight loss of fresh-cut apples with and without edible coatings was also evaluated (Figure 6). Significant weight loss occurred in uncoated fresh-cut apples (control). Constant losses in weight were observed in all coated samples up to day 7, except for samples that were coated with the bioactive edible formula 5, which exhibited no significant reduction in weight at the end of 7 days of storage. Bioactive edible formula 6 also exhibited a protection against weight loss, though not to the same degree as formula 5. Previous studies reported weight loss of stored product was lower when yeasts of the species *Cryptococcus laurentii* were added in the coatings, suggesting that the addition of microorganisms can influence this aspect of food preservation [43]. Our result also suggested the lower WVP of formula 5, compared to formula 6 (Table 3), may help to counter water loss from the product. Reduction in product weight loss and darkening, as well as maintaining the freshness and extending the shelf life of fresh-cut products, are all important factors for the commercialization of edible films. Our results indicated bioactive edible formula 5 was the best suited to meet these parameters.

**Figure 7:**
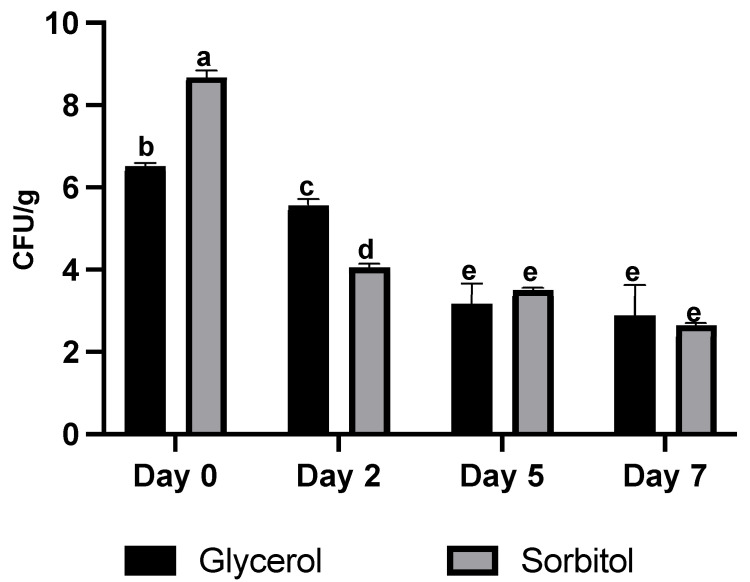
The survival rate of *E. faecium* in bioactive edible films (formula 5 and formula 6) after refrigeration at 4 °C on days 0, 2, 5 and 7. The different letters represent a significant difference of *p* < 0.05.

The global market for probiotics has greatly expanded in recent years guided by the rising demand for healthy diets and wellness. Probiotics can be delivered either through conventional means via pharmaceutical related products or non-conventional methods such as in food-based products. Probiotics have been added to several food products as well as incorporated into biopolymeric matrices to develop active food packaging. This packaging serves as an alternative method for controlling foodborne microorganisms, improving food safety, and providing health benefits [31]. In our study, probiotic *E. faecium* isolated from fermented chicken was incorporated into cactus mucilage containing either glycerol or sorbitol as a plasticizer. We evaluated the probiotic strain in films to assess stability in terms of bacterial cell viability. First, we studied the viability of the probiotic after submerging fresh-cut apple slices in solutions containing cactus mucilage and plasticizer (either glycerol or sorbitol) and the probiotic bacteria (10^6^–10^8^ CFU·g^−1^). The viability of the probiotics on the coated fresh cut apple slices dropped from the initial population of 10^6^–10^8^ CFU·g^−1^ (on Day 0) to 10^3^ CFU·g^−1^ on day 7 after refrigeration at 4 °C (Figure 7). The film formula, processing temperature for the film distribution and the storage conditions have all been found to influence the viability of the probiotic. Although there is no consensus among the international scientific community about effective probiotic doses to achieve health effects, researchers have suggested minimum doses between 10^6^ and 10^9^ CFU·g^−1^ to ensure therapeutic effects [44]. Other studies have shown probiotic viability in pure pullulan film was around 80% after 10 days of storage at 25 °C, decreasing to 35% after 20 days. Films incorporated with starches, however, show a correlation of decreased cell viability as the starch content increases [16]. Rice starch and proteins were observed to act synergistically to promote *Lactobacillus rhamnosus* GG cell viability [45]. Moreover, some factors such as WVP, oxygen content, water activity (a_w_), heat-induced injuries, types of probiotic strains and mechanical stress can have adverse effects on the viability of probiotics in films [32]. For example, the addition of fructo-oligosaccharides (FOS) as a prebiotic and polysorbate 80 as a plasticizer together with glycerol and sodium alginate in linseed mucilage have been reported to maintain the number of the probiotic *Lactobacillus casei* LC-01 for 15 days [17]. Inulin, chitosan oligosaccharide (COS), galacto-oligosaccharide (GtOS) and FOS have been added to maize starch-based edible films for prebiotic purposes and have been shown to promote the growth of the probiotic bacteria *Bifidobacterium infantis* ATCC 15697 and *Lactobacillus fermentum* ATCC 9398 [46]. We hypothesize that the improvement in cell viability in those studies might be attributed to compounds in the films that promoted cell metabolism. Additionally, the conditions of film creation/drying and the storage condition might also be crucial for improvements in probiotic viability. Further study and process improvement for the incorporation of probiotics into bioactive edible films is required in our study.

## 4. Conclusions

We developed an edible coating for fresh-cut apple slices that resulted in improved preservation and shelf life of the product. We identified cactus mucilage, gelatin, glycerol and *E. faecium* as a probiotic (formula 5) as the optimal formula for an edible film, according to parameters which analyzed physicochemical and physical properties. A number of studies in the last decade have focused on the incorporation of probiotics and other active ingredients to create bioactive edible films, leading to the emergence of new and exciting technology. From our study, the probiotic film and coatings played a significant active role in extending food stability by minimizing the weight loss and maintaining the freshness of fresh-cut apple slices. Improvements are still required for maintaining the number of surviving probiotic microbes in our edible film during food storage. Future plans for the improvement of our film will include the addition of prebiotics, the modification of the plasticizers and utilization of other probiotic strains. We hope that edible films composed of cactus mucilage and a probiotic may be a novel functional food that could replace supplement intake. Our current findings are an exciting first step toward modification of bioactive edible films/coatings for use with fresh-cut fruits.

## Figures and Tables

**Table 1:** Film formulation.

Formula No.	Distilled Water (mL)	Cactus Mucilage (mL)	Gelatin (g)	Plasticizer (mL)		Probiotic Added
				Glycerol	Sorbitol	
1	170	-	5	5.7	-	-
2	170	-	5	-	5.7	-
3	85	85	5	5.7	-	-
4	85	85	5	-	5.7	-
5	85	85	5	5.7	-	+
6	85	85	5	-	5.7	+

**Table 2:** Composition of ethanol extracted cactus mucilage.

Total Sugar (mg/g)	Phenolic Content (mg AGE/mL MUC)	Vitamin C (mg/mL)	Percent DPPH Radical Scavenging (%)
0.47 ± 0.06	0.33 ± 0.06	0.14	35.51 ± 1.88

**Table 3:** Film thickness, percent moisture content, percent solubility and water vapor permeability of the 6 film formulas. The different letters represent a significant difference of *p* < 0.05. The error bar indicates the standard deviation (*n* = 3).

Formula	Film Thickness (mm)	Moisture Content (%)	Film Solubility (%)	Water Vapor Permeability (g·mm/m^2^·min·kpa)
1 (Glycerol)	0.01 ± 0.01 ^c^	0.36 ± 0.03 ^a^	60.65 ± 3.27 ^a^	0.05 ± 0.001 ^c^
2 (Sorbitol)	0.06 ± 0.03 ^b^	0.18 ± 0.01 ^b^	40.97 ± 4.91 ^c^	0.32 ± 0.001 ^b^
3 (Glycerol + Cactus mucilage)	0.02 ± 0.02 ^bc^	0.24 ± 0.07 ^b^	59.41 ± 6.89 ^a^	0.15 ± 0.070 ^c^
4 (Sorbitol + Cactus mucilage)	0.02 ± 0.01 ^c^	0.21 ± 0.04 ^b^	45.43 ± 2.73 ^bc^	0.16 ± 0.071 ^b^
5 (Glycerol + Cactus mucilage + Probiotic)	0.02 ± 0.01 ^bc^	0.21 ± 0.05 ^b^	53.15 ± 3.79 ^ab^	0.31 ± 0.008 ^b^
6 (Sorbitol + Cactus mucilage + Probiotic)	0.11 ± 0.04 ^a^	0.19 ± 0.01 ^b^	30.66 ± 7.23 ^d^	1.50 ± 0.009 ^a^

Note: Different lowercase superscripts denote significant difference within the column (*p* < 0.05).

## Data Availability

Not applicable.
